# The Effect of Polychlorinated Biphenyls on the Song of Two Passerine Species

**DOI:** 10.1371/journal.pone.0073471

**Published:** 2013-09-18

**Authors:** Sara DeLeon, Rayko Halitschke, Ralph S. Hames, André Kessler, Timothy J. DeVoogd, André A. Dhondt

**Affiliations:** 1 Department of Ecology and Evolutionary Biology, Cornell University, Ithaca, New York, United States of America; 2 Department of Biodiversity, Earth, and Environmental Science; Drexel University, Philadelphia, Pennsylvania, United States of America; 3 Laboratory of Ornithology, Cornell University, Ithaca, New York, United States of America; 4 Department of Psychology, Cornell University, Ithaca, New York, United States of America; University of Lethbridge, Canada

## Abstract

Polychlorinated biphenyls (PCBs) are synthetic chemical pollutants with demonstrated detrimental toxic and developmental effects on humans and wildlife. Laboratory studies suggest that PCBs influence behavior due to their effects on endocrine and neurological systems, yet little is known about the behavioral consequences of sublethal PCB exposure in the field. Additionally, specific PCB congener data (in contrast to total PCB load) is necessary to understand the possible effects of PCBs in living organisms since number and position of chlorine substitution in a PCB molecule dictates the toxicity and chemical fate of individual PCB congeners. We non-lethally investigated total PCB loads, congener specific PCB profiles, and songs of black-capped chickadees (*Poecile atricapillus*) and song sparrows (*Melospiza melodia*) along a historical PCB gradient at the Hudson River in New York State. Our results indicate that black-capped chickadees and song sparrows have higher total blood PCBs in regions with higher historic PCB contamination. The two bird species varied substantially in their congener-specific PCB profiles; within sites, song sparrows showed a significantly higher proportion of lower chlorinated PCBs, while black-capped chickadees had higher proportions of highly chlorinated PCBs. In areas of PCB pollution, the species-specific identity signal in black-capped chickadee song varied significantly, while variation in song sparrow trill performance was best predicted by the mono-*ortho* PCB load. Thus, PCBs may affect song production, an important component of communication in birds. In conclusion, we suggest that the ramifications of changes in song quality for bird populations may extend the toxic effects of environmental PCB pollution.

## Introduction

Polychlorinated biphenyls (PCBs) are global chemical pollutants first manufactured in the United States in 1929 [1]. Although PCB production was banned in 1979, they continue to pose environmental risks in areas of hotspot contamination due to unsound disposal practices and chemical characteristics [1]. It is estimated that 10^8^ kg of PCBs are still present in the biosphere worldwide [2].

PCBs are a complex class of hydrophobic, lipophilic chemicals that often slowly decompose and metabolize in biological systems [3]. Their two benzene ring structure allows for ten possible positions of chlorination, resulting in 209 congeners with variable toxicities, environmental persistences, and biological effects [3]. In general, higher chlorinated PCBs are longer lasting, less likely to be metabolized, and more toxic [4]. Coplanar PCBs (unchlorinated at the 2, 2′, 6, 6′-*ortho* position) and mono-*ortho*-substituted PCBs have a flatter structure with chemical properties and toxicities similar to dioxin [4]. Some PCBs are also endocrine disruptors [5], and PCBs with fewer chlorines are associated with estrogenic actions [6], while some congeners have demonstrated androgenic or antiandrogenic activity [7].

Because the position and number of chlorines on the di-benzene backbone dictate the toxicity and chemical fate of PCB congeners, specific congener data (in contrast to total PCB concentration) is necessary to understand the possible effects of PCBs in living organisms. However, PCB profiling is often only performed on larger organisms at high trophic positions, because PCBs bioaccumulate and biomagnify [1], and because the large amount of the sample permits analysis of congener-specific PCBs (e.g. [8]). When PCBs are measured in smaller organisms at lower trophic positions, destructive sampling is commonly used to obtain samples large enough in size to determine congener-specific PCB data (e.g. [9]). In circumstances where species are protected or endangered, the alternative is to look at PCB contamination levels in carcasses [8], a practice that may result in biased sampling for PCB levels, if PCBs affect mortality. Although advances have been made in developing new methods to non-lethally measure PCB load in small amounts of wildlife blood, or feather and hair samples (e.g. [10], [11]), these methods have not yet been commonly tested or used in field studies. Therefore, our first objective was to quantify congener-specific PCB levels using small volumes of blood from individual passerines in the field.

PCB congeners are known to interfere with natural endocrine regulation [5]. Since many complex behaviors are the endpoints of neurological processes regulated by hormones, PCBs could have widespread behavioral effects [9]. Yet, few field studies support this hypothesis (but see [12]–[15]). Establishing the magnitude and extent of PCB effects on behavior could provide a framework for assessing, predicting, and mitigating the consequences of sublethal PCB exposure for wildlife populations.

Song is a critical behavior for many bird species and is used primarily for mate identification and attraction, and territory defence [16]. Song is also a behavior sensitive to chemical pollutants, such as dichlorodiphenyltrichloroethane (DDT) [17], heavy metals [18], natural and synthetic estrogen mimics [19], and 2,2′,4,4′,5-pentabromodiphenyl ether (BDE-99) [20]. However, the effects of PCBs on birdsong is not known. PCBs may be influencing song phenotypes by disrupting androgenic and estrogenic metabolism of testosterone important in regulating the song control nuclei in the passerine brain (reviewed in [21]). In laboratory experiments, PCB exposure has been shown to cause a decrease in volume of the robust nucleus of the arcopallium (RA), a motor song region in bird brains [22]. Since the volume of the RA is correlated with aspects of song structure [23], PCBs could be directly influencing the neural basis of song characteristics. Therefore, our second objective was to ask whether song characteristics covary with PCB levels along a contamination axis. Because PCBs have toxic [4] as well as endocrine [5] effects, we made no directional prediction on the effects of PCBs on birdsong, but simply hypothesized that song would differ between birds exposed to higher levels of PCB pollution and birds with lower levels of PCB-exposure.

Focusing on black-capped chickadees (*Poecile atricapillus*) and song sparrows (*Melospiza melodia*) along a PCB gradient in northeastern New York State, USA, we investigated whether sublethal levels of PCB exposure affect biologically relevant birdsong characteristics in these populations. Our main objectives were to non-lethally sample blood from black-capped chickadees and song sparrows in the field to quantify congener-specific PCB levels and to assess birdsong characteristics as a function of PCB exposure. To account for additional environmental and developmental factors that may influence birdsong characteristics in these populations, we also quantified body condition of the two species [24], [25] and blood mercury concentrations [26].

## Materials and Methods

### Study sites

In 1991 the Environmental Protection Agency (EPA) reported that General Electric (GE) plants at Fort Edward and Hudson Falls illegally discharged up to 1.3 million pounds of PCBs into the Hudson River between 1947 and 1977 [27]. Today, the Hudson River basin continues to contain high PCB levels that vary throughout the drainage.

**Figure 1:**
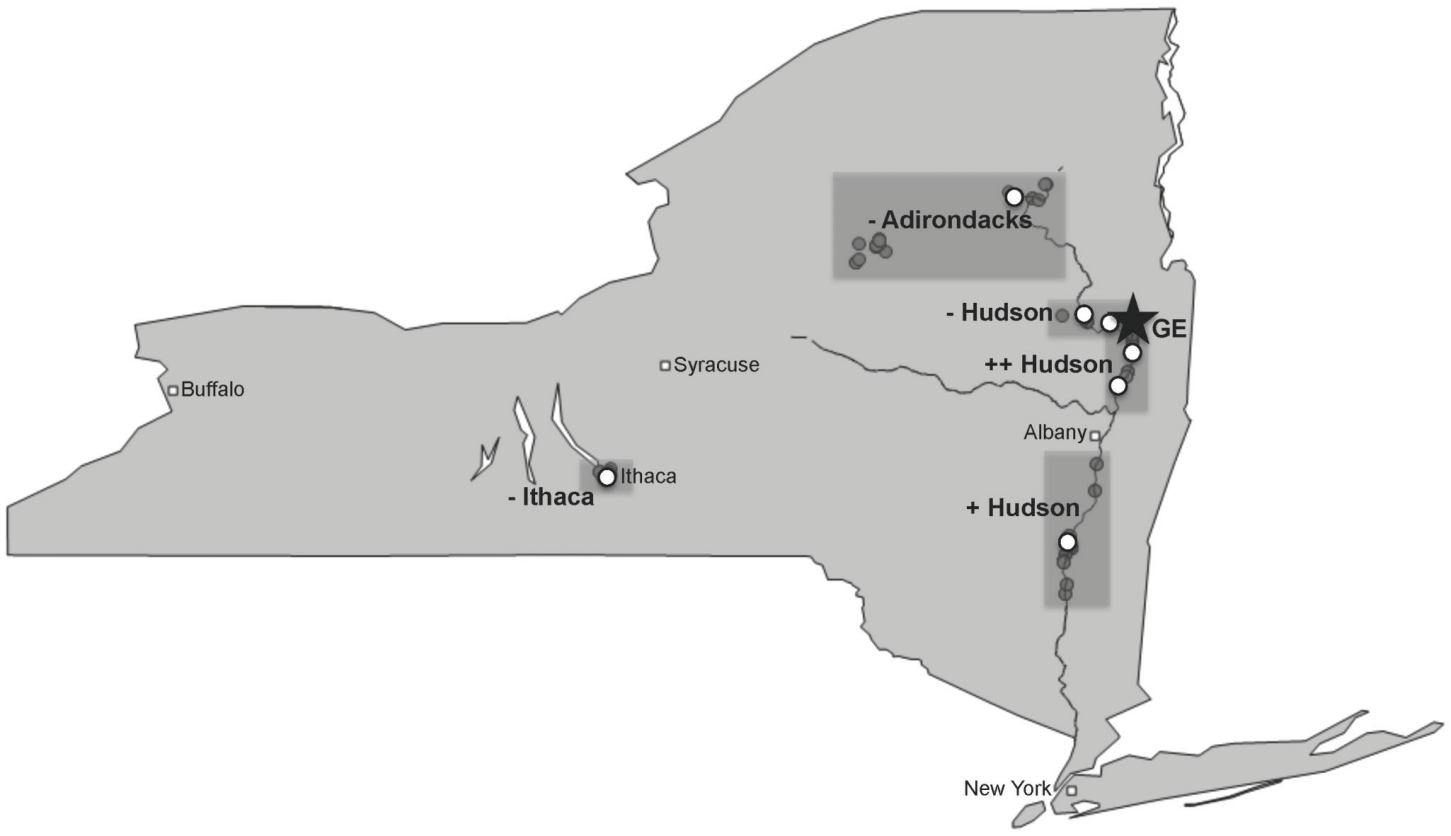
Map of recording and mist-netting sites. The five study regions are indicated by grey rectangles. − Ithaca is in Ithaca, NY. − Adirondacks is in the Adirondack Mountains. − Hudson is directly above the point source pollution at General Electric (GE, indicated by a star), + Hudson and ++ Hudson are downstream of GE. Recording sites are indicated with dark grey points, and mist-netting sites are indicated with white points.

From 2006 to 2009, 48 recording sites and 7 mist-netting sites were established in five regions in New York State (Figure 1). These regions were chosen based on the documented historic variability in the levels of PCBs found in the Hudson River basin [28]. Three regions were identified with no local point source PCB inputs (labelled as − Ithaca, − Adirondacks, − Hudson), and two regions were identified with varying environmental historic PCB inputs (labelled as + Hudson and ++ Hudson) [28]. − Ithaca was located in Ithaca, New York (N = 5 recording sites, N = 1 mist-netting site), − Adirondacks was located in Adirondack Park, New York (N = 14 recording sites, N = 1 mist-netting site), and − Hudson was located above the point source of PCB inputs on the Hudson River (N = 5 recording sites, N = 2 mist-netting sites) [28]. The region downstream from the point source of PCB inputs, with historically intermediate PCB levels, was labelled + Hudson (N = 12 recording sites, N = 1 mist-netting site) [28]. The region directly below the pollution point source, with the highest historical PCB level, was labelled ++ Hudson (N = 12 recording sites, N = 2 mist-netting sites) [28]. The sites at − Ithaca and − Adirondacks were within 40 m of a river or lakeshore, and the sites at − Hudson, + Hudson, and ++ Hudson were within 40 m of the Hudson River. All sites were in or on the edge of mixed deciduous/evergreen forests, with similar amounts of tree cover.

### Study species

We used black-capped chickadees and song sparrows as study species because both have life history traits and song characteristics that make them particularly vulnerable to PCB exposure. Adults feed their young insects and also primarily eat insects themselves during the breeding season [29]–[32]. Both sexes have relatively short natal dispersal distances, with males dispersing shorter distances than females [29], [30], [33], [34]. Additonally, both species have territories within foraging distance of water and are either year-round residents or have high between-year site fidelity [29], [30], [35], [36]. Because insects with an aquatic larval stage have higher amounts of pollutants if collected near a pollution source [37], it is reasonable to infer that birds hatched in areas with high PCBs are likely to be ingesting them for their entire life. In addition, black-capped chickadees and song sparrows both have well-studied songs and researchers have identified specific song characteristics important in communication. In this study the glissando and interval ratios were measured in male black-capped chickadee songs (Figure S1, [38]), and the trill-rate frequency-bandwidth trade-off was measured in male song sparrows (Figure S2, [39]).

### Subjects

In May and June 2008–09, black-capped chickadees and song sparrows were captured using 3 m or 6 m 30 mm mist nests (Avinet, Inc. Dryden, NY 13053 USA; USFWS bird banding permit 22669: André A. Dhondt). To attract birds to the mist nets, birdfeeders with sunflower seeds were hung near net locations. Playbacks with recordings from the target species were played directly adjacent to the nets to attract territorial males. The playbacks were all songs from wild individuals recorded during the two previous field seasons (2006–07) at comparable sites. All sampling and field measurements were performed on birds within 20 minutes of capture, individuals were released directly following the procedure, and all efforts were made to minimize distress. All bird-handling procedures were approved under the Institutional Animal Care and Use Committee Protocol 2008-0022 of Cornell University.

Species were identified and sexed according to Pyle [40], and then banded with metal USFWS bands. Wing chord, tarsus length, weight, muscle score (score from 0–5 of the size of the pectoral muscle), fat score (score from 0–3 of the fat stores in the furcula) and mites (score from 0–3 of ectoparasite damage on the primary wing feathers) were recorded.

Blood samples were collected, when possible, from all individuals. Approximately 100 µL of whole blood was taken from the left brachial vein with a 27-gauge single-use needle (BD Medical, 1 Becton Dr., Franklin Lakes, NJ 07417 USA; length: 12.7 mm). The blood samples for PCB analysis were collected with a non-heparinized capillary tube and immediately transferred to 1.5 mL centrifuge tubes (EDTA washed to prevent coagulation) and stored on ice. PCB samples were separated into red blood cells and plasma with a Mini Centrifuge (Fisher Scientific, Inc., 2000 Park Lane Dr., Pittsburgh, PA 15275 USA) in the field, flash frozen on dry ice or liquid nitrogen, and stored at −80°C for later analysis. The blood samples for mercury analysis were collected with a heparinized capillary tube, stored briefly on ice, and then transferred to a −20°C freezer until analysis.

### PCB blood analysis

Blood serum samples from black-capped chickadees (22.1±2.2 mg) and song sparrows (50.6±2.2 mg) were fortified with 25 µL of ^13^C_12_-labeled internal standard mixture (EC-5087; Cambridge Isotope Laboratories, Inc., Andover, MA 01810 USA). Samples were extracted twice with 100 µL of n-hexane as described by Rivera-Rodriguez et al. [10]. The hexane extracts were dried over 100 mg anhydrous sodium sulphate and the volume reduced to 50 µL under a gentle stream of nitrogen. PCB levels were determined by gas chromatography mass spectrometry (GCMS) on a Varian Saturn 2200 ion trap GCMS system (Varian, Inc., Walnut Creek, CA 94598 USA). One µL aliquots were injected into the GC at an injector temperature of 300°C. PCBs were separated on a VF-5MS capillary column (30 m×0.25 mm ID, 0.25 micron film thickness; Varian, Lake Forest, CA 92630 USA) with helium as carrier gas at a flow rate of 1 mL min^−1^ and the following temperature gradient: 40°C for 1 min, at 30°C min^−1^ to 160°C, at 4°C min^−1^ to 220°C, at 12°C to 300°C min^−1^, and 300°C for 5 min. Congener-specific selected reaction monitoring (SRM) conditions were optimized for the quantitation of 41 of the 48 most abundant congeners reported for the Hudson River Superfund Site with quantitation limits of 0.1 to 1 ppb (Table S1C, [41]). Calibration curves were generated using a dilution series made from pure PCB standards (AccuStandard, Inc., New Haven, CT 06513 USA).

### Mercury blood analysis

A large enough blood sample to measure both mercury and PCB content could only be collected from song sparrows. Blood samples collected in 2008 from song sparrows were analyzed for total mercury content (THg) at the Centre for Environmental Sciences and Engineering (University of Connecticut, Storrs, CT 06269 USA) using Environmental Protection Agency Method 1631. Blood was first digested with sulphuric and nitric acids, oxidized using bromine monochloride, and trapped on a gold trap. The trap was purged into a cold vapour atomic fluorescence (CVAFS) unit for analysis. Detection limit for blood THg was 0.02 ng/g. Standard quality assurance methods were used: analysis of duplicate samples; method blanks; spiked samples; laboratory control samples; and standard reference materials (DOLT-3, DORM-2, 966). Instrument response was evaluated using calibration verification standard and blank before, during (every 20 samples), and after each analytic run.

### Song recording

All recordings were made with a Fostex FR-2 Field Memory Recorder (Fostex Company, 3-2-35 Musashino, Akishima, Tokyo, Japan 196-0021) or a Tascam HD-P2 Recorder (TEAC Corporation, Nakacho, Musashino-shi, Tokyo 180-8550, Japan), a Universal Telinga Pro 24-inch Parabola (Telinga Microphones, PI. 129 Botarbo, S-748 96 Tobo, Sweden), and a Sennheiser ME62 Omni Microphone (Sennheiser Electronic Corporation, 1 Enterprise Drive, Old Lyme, CT 06371, USA). Recordings were performed with a sampling width of 24 bits and a sampling rate of 44.1 kHz. All songs were recorded between 0400 and 1000 EST.

Recordings were made of spontaneous male black-capped chickadee and song sparrow song. To increase the likelihood of non-repeated sampling, individuals were distinguished by either visual or auditory cues. If there still was uncertainty of replication, the next recording was made at least one territory's distance away (∼160 m). In 2009, a subset of the song sparrows from which we obtained blood samples were also color banded and recorded (N = 29).

### Song analysis

The familiar *fee-bee* song (Figure S1) is usually only sung by male black-capped chickadees to defend a territory and to attract and arouse females during the breeding season [42]. The glissando ratio (see Figure S1) is thought to indicate species identity and varies <2% over the species range [38], [43]. The interval ratio (see Figure S1) is thought to indicate male quality [43]. High quality males are able to reproduce both ratios consistently at all frequencies (see Figure S3), and the ratios vary <2% over their range [43].

Although black-capped chickadee songs were recorded from April–August (2006–07), the recordings used in the analysis were limited to recordings made in May through early July (2006–07), while adults in our populations were breeding and seasonal song variation was at a minimum. Only recordings of black-capped chickadees singing at least eight consecutive non pitch-shifted *fee-bee* songs were used in this analysis (glissando ratio: N = 210; interval ratio: N = 218). Spectrograms were generated in RavenPro 1.4 (Bioacoustics Research Program, Cornell University, Ithaca, NY 14850 USA) with a 512 sample size, a 512 DFT size, spectral overlap of 50% (Hann window), 3 dB filter bandwidth at 135 Hz, 256 sample hop size, grid spacing of 93.8 Hz, no clipping, and averaging 1 spectrum. The glissando ratio was calculated by dividing the 95% *fee* frequency by the 5% *fee* frequency. The 95% *fee* frequency is the frequency value for a selected *fee* note where 95% of the energy is below that value. Similarly the 5% *fee* frequency is the frequency value for a *fee* note where 5% of the energy is below that value. These two measurements calculated by RavenPro give a stereotyped frequency value of the beginning *fee* note frequency and the end *fee* note frequency. The interval ratio was calculated by dividing the center frequency of the end of the *fee* note by the center frequency at the beginning of the *bee* note. Because a stereotyped measurement was not possible to use for the interval ratio, interval ratio measurements were performed manually, while blind to the source of the song. To look at the variation of the song structure within an individual black-capped chickadee, the coefficient of variation (CV) of the glissando ratio and the interval ratio was calculated for each individual. We conservatively classified individuals as having inconsistent (hereafter ‘variable’) songs if they had a glissando ratio or interval ratio CV value greater than 0.03, and as having consistent (hereafter ‘stereotyped’) songs if the CV was less than 0.03. We choose 0.03 as the cut-off point because it resulted in the most conservative grouping; only individuals singing with extremely high variation were classified as variable singers. We statistically analyzed the proportion of variable singers in a population because, although we expected variation in the song of each individual, we were especially interested in the variation between regions of outlier singers with especially high CVs.

Song sparrows are known for their melodic and varied songs. Territorial males typically sing a repertoire of approximately 4–12 song types (Figure S2) during the breeding season [44]. Although large repertoire size has been linked to large territory size, longer territory retention, and higher lifetime reproductive success [45], accurate estimates of repertoire size are difficult to obtain (minimum of 200 recorded songs needed [44]). Therefore, in this study we classified song sparrow song performance as the trill-rate frequency-bandwidth trade-off [39]. The trill is a phrase present in most song sparrow song types, characterized by the rapid repetition of a syllable. The trill rate is the number of syllables produced per unit time (Hz), and the frequency bandwidth is the difference between the maximum and minimum frequencies of the syllables in the trill. The bird is constrained in how quickly it is able to transition between low frequency production (with a relatively closed beak and a fully inflated nasopharyngeal cavity) and high frequency production (with a relatively open beak and an only partially inflated nasopharyngeal cavity), resulting in the observed trill-rate frequency-bandwidth trade-off [39].

Song sparrow songs used in the analysis were recorded from April to August in 2006, 2007, and 2009. Song sparrows that sang song types containing a trill were included in the analysis if they sang a minimum of five repetitions of the trill, and if the trill contained a minimum five syllable repetitions (N = 155). Many male song sparrows were recorded singing multiple trill types. One trill type was randomly chosen for each male to avoid pseudoreplication. Spectrograms were generated in RavenPro 1.4 (Bioacoustics Research Program, Cornell University, Ithaca, NY 14850 USA) with a 557 sample size, a 1024 DFT size, spectral overlap of 80.1% (Hann window), 3 dB filter bandwidth at 124 Hz, 111 sample hop size, grid spacing of 46.9 Hz, no clipping, and averaging 1 spectrum. The trill rate of a trill of n syllables was calculated by dividing the count of n−1 syllables by the time span of n−1 syllables (to account for inter-syllable time). The bandwidth was measured by subtracting the 0.5% frequency from the 99.5% frequency and averaged for at least five syllables per trill. Trills were binned into 5-Hz trill rate increments and from each bin the trill with the maximum frequency bandwidth was selected and used to produce the linear regression as described in Podos 1997 [39]. We classified individuals singing trills with a trill rate and a bandwidth that fell less than 4 kHz from the regression line as singing high performance trills and individuals singing trills with a trill rate and a bandwidth that fell more than 4 kHz from the regression line as singing low performance trills. Four kHz was chosen as the dividing point because it resulted in the most conservative grouping; only individuals singing trills with extremely low performances were classified as low performance singers. We statistically analyzed trill performance as a dichotomous variable because, although we expected variation in trill performance of song in each individual, we were especially interested in the variation between regions of outlier singers with especially low trill performance. Trill types varied greatly within and between regions. However, no attempt was made to classify the trill types, and all trills were pooled for analysis. This greatly increased the noise of the data, decreasing the likelihood of finding any pattern, and therefore making this a conservative analytical approach.

### Statistical analysis

Total PCB data were log-transformed and analyzed with a two-way ANOVA in which species identity and region were fixed effects, followed by a Tukey post-hoc test. The difference in total PCB concentration between sexes and age classes were analyzed with an ANOVA with sex and age class as fixed effects. Congener-specific PCB data were analyzed in the ++ Hudson region with a principal components analysis (PCA). The proportions of tetra- and penta-chloro (4-Cl+5-Cl), and hepta- and octa-chloro (7-Cl+8-Cl) PCBs in the ++ Hudson were compared separately between the two species with Student's t-tests.

The regional differences of black-capped chickadee glissando and interval ratios were analyzed with an ANOVA with region as a fixed effect, and significant differences were followed with a Tukey post-hoc test. The average glissando and interval ratios in each region were compared to ratios published in Christie *et al.* [43]. A Chi-square test was used to assess whether proportions of stereotypical and variable individual black-capped chickadee singers differed between regions.

In the song sparrow analysis, a Chi-square test was used to test whether proportions of individual song sparrows singing high performance and low performance trills differed between regions. In the 2009 subset of song sparrows in which individual males were sampled for song, location of the individual, PCB profile, and body condition, Generalized Linear Models (GLMs) were built and ranked using the Akaike Information Criterion corrected for small sample sizes (AICc).

Body condition variables and blood mercury concentrations were analyzed with an ANOVA with region as a fixed effect, and Tukey post-hoc tests.

## Results

### PCB concentrations in black-capped chickadee and song sparrow blood

Analysis of PCB profiles in the blood of black-capped chickadees and song sparrows along the contamination gradient in northeastern New York (Figure 1) revealed region- and species-dependent differences in PCB concentration and congener composition. The average concentration of the 41 congeners analyzed for black-capped chickadees (Table S1A) and song sparrows (Table S1B) varied greatly among the five regions and between species, but not between sexes or age classes (Black-capped chickadees: 74% male, ANOVA: F = 1.31, *P* = 0.29; Song sparrows: 90% male, ANOVA: F = 1.39, *P* = 0.25).

**Figure 2:**
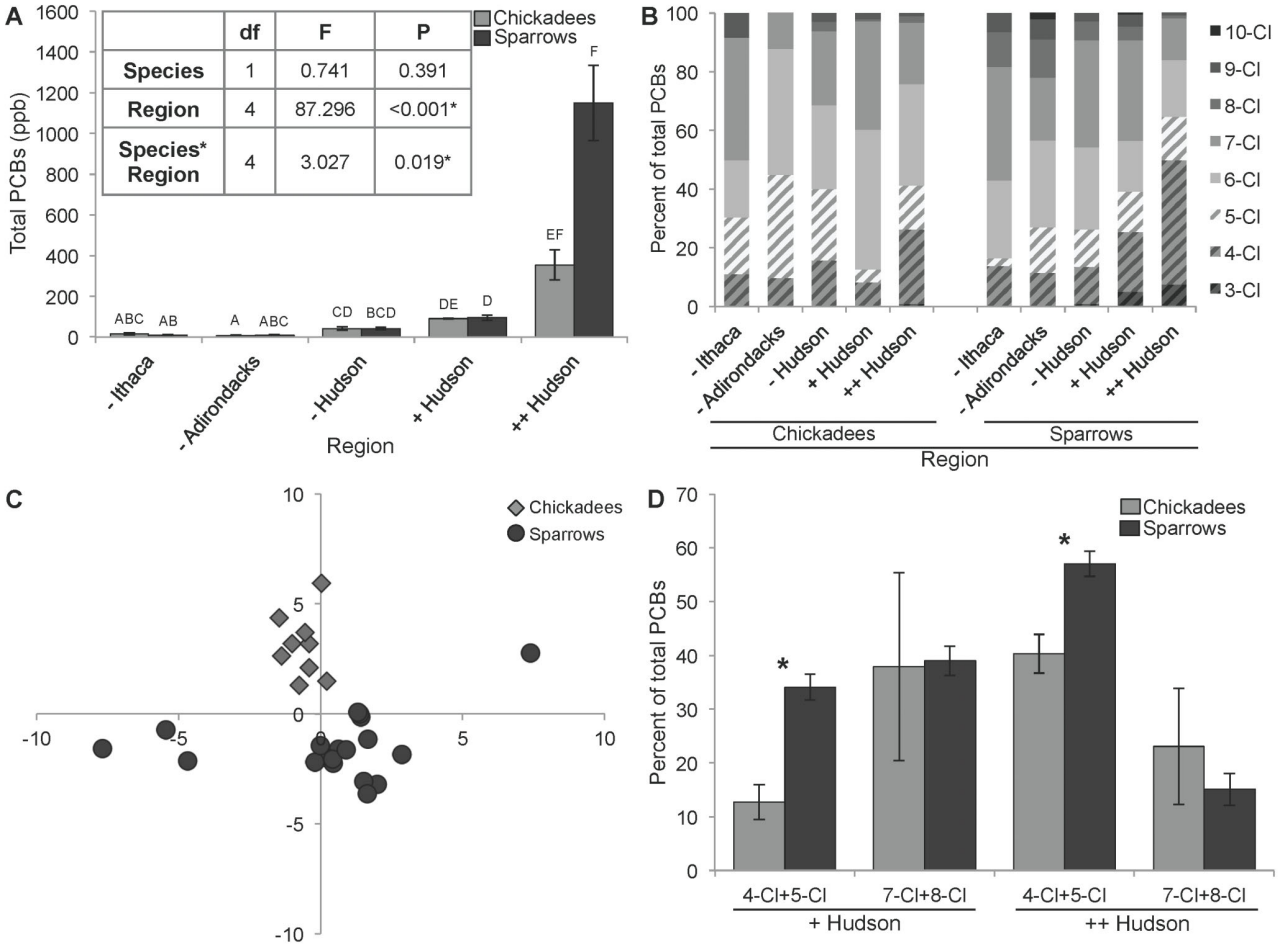
Differential bioaccumulation of PCBs in two songbirds. **A**) Total PCB concentration (ppb, mean±SE) were analyzed in blood samples of black-capped chickadees and song sparrows from northeastern New York State. ANOVA (inset) was performed on log-transformed data. Non-overlapping letters indicate statistical difference between groups. **B**) The PCB chlorination profiles for black-capped chickadees and song sparrows by region. **C**) Principal component analysis of PCB congener profiles from black-capped chickadees and song sparrows from the region with highest historical PCB contamination (++ Hudson). PC1 accounts for 21% of variability, and PC2 accounts for 19.3% of variability. **D**) The proportion of lower chlorinated (4-Cl+5-Cl) and higher chlorinated (7-Cl+8-Cl) PCBs in black-capped chickadees and song sparrows from the + Hudson and ++ Hudson regions.

The black-capped chickadee total PCB concentration in the ++ Hudson region was higher, but not significantly so, than total PCB concentrations found in individuals from the + Hudson region (Figure 2A). Chickadees in the − Ithaca, − Adirondack, and − Hudson regions had significantly lower total PCB concentrations than individuals from the ++ Hudson regions (Figure 2A). The PCB congener profile in black-capped chickadees varied between regions, and chickadees in the + Hudson region appeared to have the largest proportion of higher chlorinated PCBs than in all other regions (Figure 2B).

Song sparrows had significantly higher total PCB concentrations in the ++ Hudson region (the area of highest historic PCB contamination [28]) when compared to all other regions (Figure 2A). While the total PCB concentrations in birds from the + Hudson and − Hudson regions did not differ significantly from each other, the total PCB concentration in song sparrows from the + Hudson region were significantly higher than the PCB concentrations found in song sparrows from the − Ithaca and − Adirondacks regions (Figure 2A). The PCB congener profile in song sparrows varied between regions, and song sparrows in the ++ Hudson region appear to have the largest proportion of lower chlorinated PCBs than in all other regions (Figure 2B).

In regions − Ithaca and − Hudson, the two bird species had a similar congener profile (Figure 2B). In region − Adirondacks, chickadees had higher penta- and hexa- chlorinated PCB levels, while song sparrows had more evenly distributed levels of PCB chlorination (Figure 2B). The greatest difference in PCB content of the two species was in the ++ Hudson region, directly below the point source pollution from the GE plant. To examine the different congener profiles between the chickadees and sparrows in the ++ Hudson region in more detail, a PCA was performed on the PCB congener data (Figure 2C). The difference in congener profiles for chickadees and sparrows partitioned clearly along PC2, which corresponds to the proportion of congeners with a lower degree of chlorination based on factor loading. In our sample from the ++ Hudson region, chickadees and song sparrows did not differ significantly in proportion of hepta- and octa- chlorinated congeners in their PCB load (Figure 2D; t = 1.30, *P* = 0.20), while song sparrows had a significantly higher proportion of lower chlorinated PCBs (4-Cl+5-Cl) than chickadees (Figure 2D; t = −3.36, *P* = 0.0016). Similarly, the proportion of lower chlorinated PCBs was higher in song sparrows in the + Hudson region, further downstream from the point source of pollution (Figure 2D; t = −2.66, *P* = 0.01).

### Black-capped chickadee song

Male black-capped chickadees are known to have a highly stereotyped song, with the glissando and interval ratios varying less than 2% across their entire North American range, according to published values (Figure S1, Table S2, [43]). Yet in our study regions, the glissando ratio varied more than 2% from published values in both the + Hudson and the ++ Hudson regions, and the interval ratio varied slightly more than 2% from the published value in the − Hudson region (Table S2, [43]).

**Figure 3:**
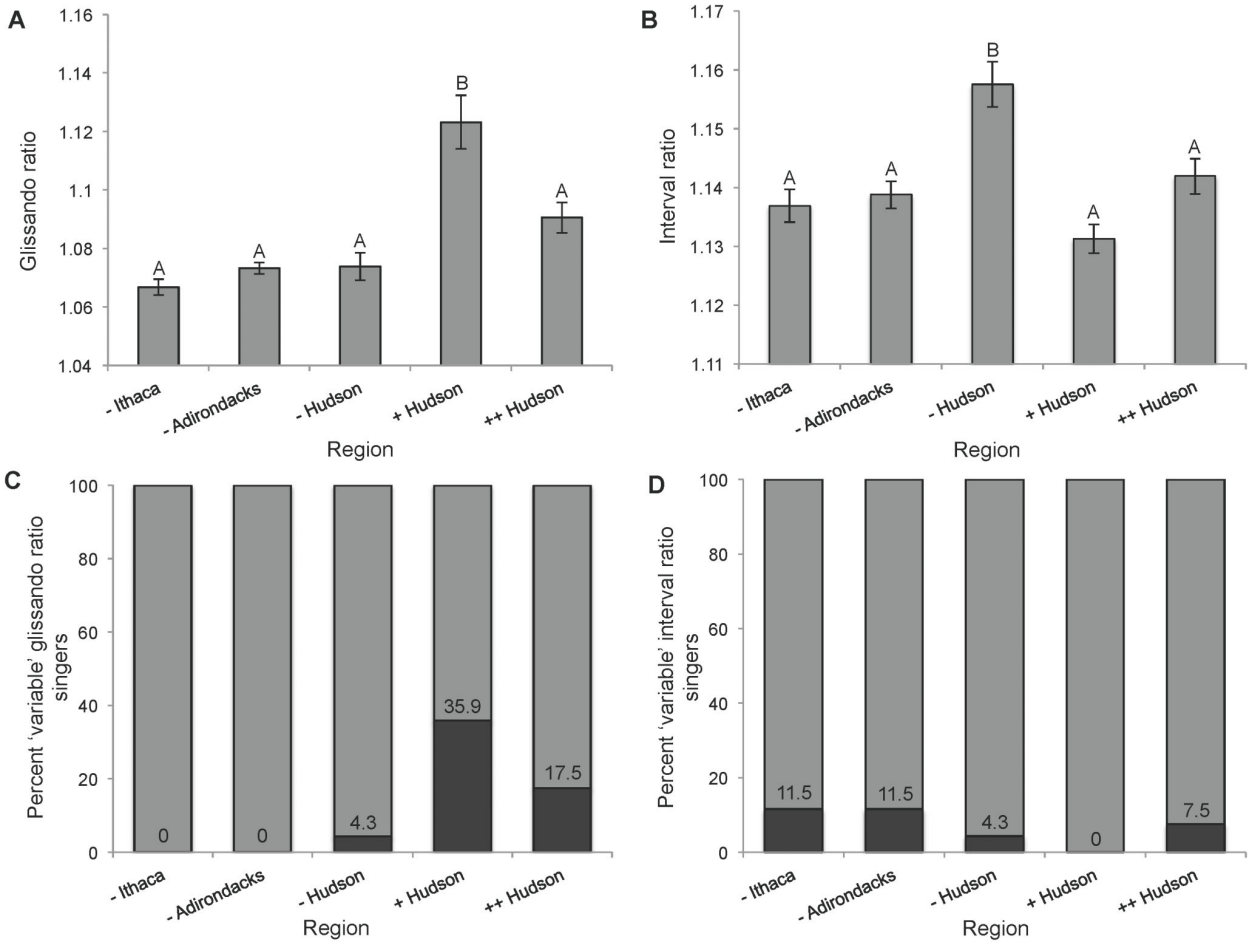
Black-capped chickadees had more variable glissando ratios in regions of PCB contamination. Average glissando ratio (**A**) and average interval ratio (**B**) of black-capped chickadees by region. Bars represent mean±SE and non-overlapping letters indicate statistical difference between regions. Proportion of stereotyped/variable glissando ratios (**C**) and interval ratios (**D**) of individual black-capped chickadees. Individuals singing stereotyped ratios (grey, CV<0.03) and variable ratios (black, CV>0.03, numbers in bars) are shown by region.

Birds from + Hudson had a significantly higher glissando ratio than birds from all other regions (Figure 3A; ANOVA: F = 9.78, *P*<0.001). Birds from the − Hudson had a significantly higher interval ratio than birds from all other regions (Figure 3B; ANOVA: F = 6.53, *P*<0.001). Additionally, individual black-capped chickadees within a region varied in the stereotypy of their glissando ratios (see Figure S3 for examples), but not in the stereotypy of their interval ratios. The proportion of individual chickadees singing inconsistent glissando differed between regions (Figure 3C; Chi-square = 42.54, df = 4, *P*<0.001). The proportion of individual chickadees singing inconsistent interval ratios was not significantly different between regions (Figure 3D; Chi-square = 7.23, df = 4, *P* = 0.12).

### Song sparrow song

**Figure 4:**
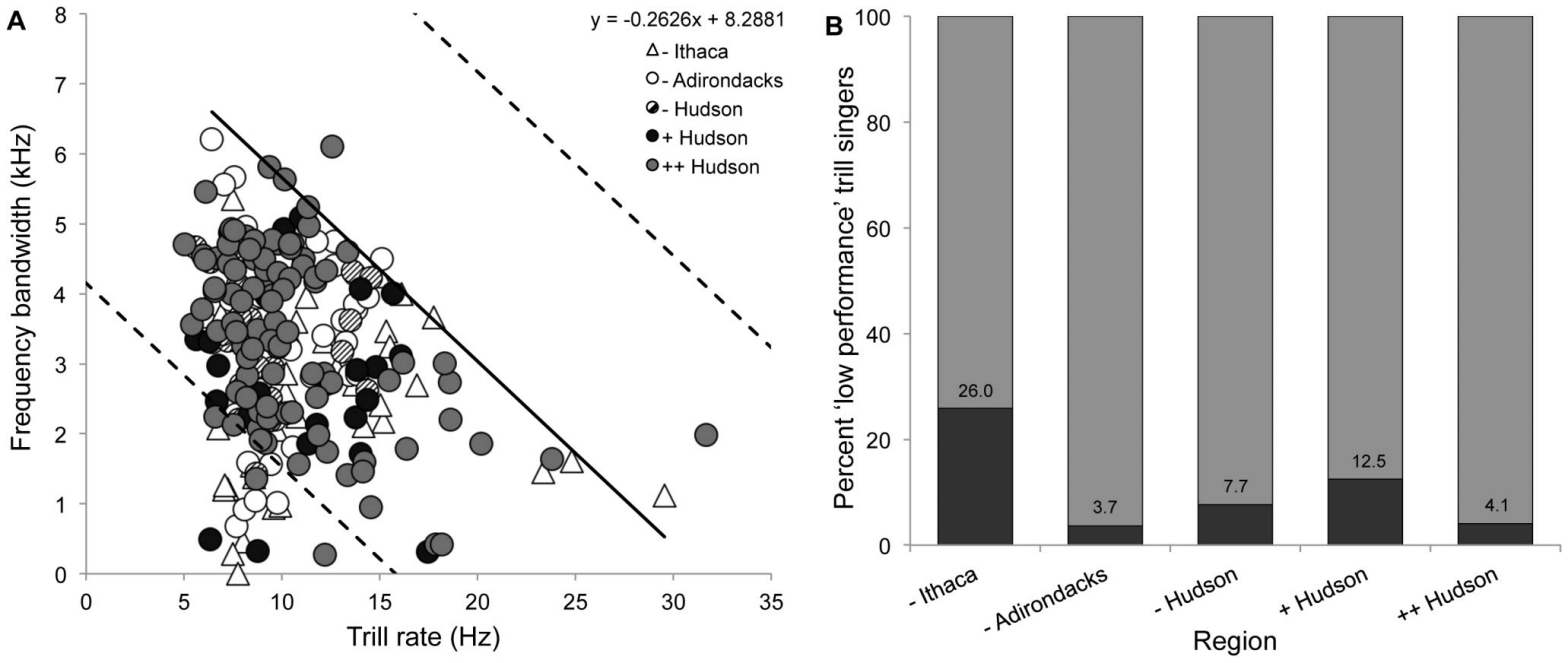
Song sparrow trill performance varied between study regions. **A**) Trill rate-frequency bandwidth trade-off of song sparrows. The regression line is calculated from all recorded trills, and each point represents a randomly chosen trill type of one individual song sparrow. The dotted lines represent the trill rate-bandwidth cut-off of 4 kHz distance from the trill performance regression line (solid). Low performance trills are between the origin and the lower dotted line. **B**) The proportion of song sparrows singing high performance (grey, distance from regression line<4 kHz) or low performance (black, distance from regression line>4 kHz) trills by region. The numbers in bars are the actual percentages of individual sparrows singing low performance trills.

All trills recorded from male song sparrows during 2006–07 were plotted as trill rate (Hz) versus frequency bandwidth (kHz), with the upper-bound regression line indicating the trill performance limit (Figure 4A, [39]). The proportion of song sparrows that sang high performance trills were significantly different between regions (Figure 4B; Chi Square = 12.80, df = 4, *P* = 0.01).

In 2009 a subset of song sparrows were sampled for blood PCB loads and body condition, and their songs were recorded. Table 1 shows the GLMs that were built and their corresponding AIC-based weight of support from the data. The variation in the average trill performance for an individual male was best described by the individuals' level of mono-*ortho* PCBs. The best-supported model contained only mono-*ortho* PCB concentrations as a predictor, and this model had approximately twice the support of the second strongest model that included both the level of mono-*ortho* PCBs and a body condition index (tarsus/mass). Although inclusion of body condition creates a model with half the support of the best model, body condition does not appear to be an independent or strong predictor of trill rate: the model with body condition index alone was the third-lowest ranked model and had almost no support from the data. Additionally, our analyses show that the effect of PCB concentrations on trill rate is not mediated by body condition. The model with an interaction between the effects of mono-*ortho* PCB level and body condition has little support from the data, with only 11% of the support of the best model. Latitude was also a poor predictor of trill performance, with only 3% of the support of the best model.

**Table 1:** AIC_c_ values and Akaike model weights for average trill performance of 2009 song sparrows.

	Variables									
Response Variable	mono-ortho PCBs	total PCBs	BCI^1^	latitude	mono-ortho PCBs*BCI^1^	total PCBs*BCI^1^	AICc	Δ AIC	Akaike weight	ratio
Average trill performance	X						89.8478	0	0.5516	1
Average trill performance	X		X				91.3580	1.5102	0.2592	0.4700
Average trill performance	X		X		X		94.2431	4.3953	0.0613	0.1111
Average trill performance		X					94.4163	4.5685	0.0562	0.1019
Average trill performance		X	X				95.7008	5.8530	0.0296	0.0536
Average trill performance			X				96.6236	6.7758	0.0186	0.0338
Average trill performance				X			96.8473	6.9995	0.0167	0.0302
Average trill performance		X	X			X	98.6291	8.7813	0.0068	0.0124

1 BCI = tarsus length/mass.

### Body condition and mercury concentration

Black-capped chickadees captured from the five regions showed no differences in wing chord length, body weight, muscle score, fat score, or feather ectoparasite damage (ANOVA: *P*>0.05), but differed in tarsus length (ANOVA: F = 3.751, *P* = 0.0141), with ++ Hudson birds having significantly longer tarsi than individuals from − Adirondacks and − Hudson (Tukey's test: *P*<0.05). Song sparrows captured from the five regions showed no differences in wing chord length, tarsus length, body weight, or feather ectoparasite damage (ANOVA: P>0.05), but differed in muscle score (ANOVA: F = 3.63, P = 0.0079), with ++ Hudson birds having significantly higher muscle scores than + Hudson' birds (Tukey's post hoc test: P<0.05). Sparrows also differed in their fat score (ANOVA: F = 5.286, P = 0.0006), with + Hudson birds having higher fat scores than − Adirondacks and ++ Hudson birds (Tukey's post hoc: P<0.05). Song sparrows from − Adirondacks had higher blood mercury concentrations than song sparrows from all other regions except − Hudson (Figure S4; ANOVA: F = 6.58, P = 0.0025).

## Discussion

The results of this field study show that the historical contamination of the Hudson River is reaching the terrestrial bird populations of black-capped chickadees and song sparrows and suggests that PCB-exposure may be affecting male song characteristics important in communication. Both black-capped chickadees and song sparrows have elevated total PCB concentrations in the ++ Hudson region, the region with the highest historical PCB contamination (Figure 2A, [28]). The total PCB concentrations found in black-capped chickadees and song sparrows captured along the historically contaminated Hudson River were comparable to previously reported values in tree swallows (*Tachycineta bicolor*) from the same region [46].

Although total PCB concentrations did not differ between black-capped chickadees and song sparrows in any area (Figure 2A), the specific PCB congener composition found in the two species differed significantly (Figure 2B–D). This disparity is likely due to different habitat and foraging preferences. Although both species predominantly eat insects during the breeding season [29]–[32], song sparrows are more commonly a riparian species that have territories and nesting sites close to the Hudson River and regularly forage around and in water [30], [35]. In contrast, black-capped chickadees are common inhabitants of nearby woodlands and forage higher in the canopy [29], [36]. The PCB profile of song sparrow blood more closely resembled the PCB congener profile found on the Hudson River [28], [41] and song sparrows had a larger proportion of lower chlorinated PCBs in the ++ Hudson region than in any other region (Figure 2B). The black-capped chickadee PCB congener profile contained more highly chlorinated PCBs, especially in the + Hudson region (Figure 2B). Overall, both species appear to have a greater proportion of higher chlorinated PCBs in regions further away from the PCB source input at GE (Figure 2B). This is likely a result of differential bioaccumulation and decomposition and could have far reaching behavioral consequences [4].

In black-capped chickadee song, individual males that reproduce consistent glissando and interval ratios within bouts of singing are dominant, high quality males that reliably broadcast their species identity [38], [43]. Although we found significant differences between regions in glissando and interval ratios (Figure 3A, B), only three regions differed more than 2% from published values (Table S2, [43]), indicating a potential biologically significant deviation of communication signals in these regions. We observed the highest variation from published species-specific values for black-capped chickadee glissando ratio [43] in the two regions with the highest PCB concentration; + Hudson region (>6% deviation) and ++ Hudson regions (>3% deviation) (Figure 3A, Table S2). In contrast, the glissando ratio showed only minor differences from published values [43] in the − Ithaca (1.02%), − Adirondacks (1.63%), and − Hudson (1.70%) regions (Table S2). Black-capped chickadee interval ratio was only significantly higher in the − Hudson region (Figure 3B), but the difference was only slightly more than 2% different from published values (2.07%, Table S2, [43]).

Similarly, only the stereotypy of the glissando ratio differed between regions (Figure 3C), but the stereotypy of the interval ratio did not (Figure 3D). Taken together, these results likely indicate a biologically significant deviation of the species identity signal (the glissando ratio) in the + Hudson region, and possibly in the ++ Hudson region as well. This deviation is not likely a result of seasonal variation, since the black-capped chickadee song sample was limited temporally to the breeding season. Likewise, the deviation is likely not a result of geographical variation, since the + Hudson and − Ithaca regions are at similar latitude, but black-capped chickadee song characteristics differ greatly between these regions (Figure 3). Therefore, since the + Hudson and ++ Hudson are regions of PCB point source pollution, and since black-capped chickadees in these regions have elevated blood PCB levels (Figure 2A), PCBs may be affecting the songs of chickadees in these regions. Interestingly, it is the black-capped chickadees in the + Hudson region with the strongest song differences, not the chickadees in the ++ Hudson region, were the PCB input was higher [28]. A potential explanation is that the effect of PCBs on black-capped chickadee song is a non-linear dose-response relationship. This observation is consistent with a U-shaped dose response curve, where the maximum response is at low and high exposure, a phenomenon known to be characteristic of endocrine disrupting chemicals [47]. Potentially, the PCB congener profile difference (Figure 2B) offers an alternative mechanism behind the variation in black-capped chickadee song in the higher PCB contaminated regions along the Hudson River.

The trill performance of song sparrows along the Hudson River was comparable to previously published values for the species (Figure 4A, [39]). The percent of high and low trill performers did vary by region (Figure 4B), indicating that song sparrow trill performance may be affected by PCB pollution. Analysis using GLMs with AIC ranking indicates that mono-*ortho* PCB loads best predict the variation in individual song sparrow trill performance (Table 1). Models using total PCB concentration had 10% of the support of equivalent models with mono-*ortho* PCBs, emphasizing the need for PCB congener quantification. The model using latitude had 3% of the support of the best model, indicating that the patterns in trill performance are not a result of geographical variation. The addition of information on body condition decreased the support of the mono-*ortho* model, as did adding the interaction term between mono-*ortho* PCB load and body condition. Taken together, these results suggest that PCB-exposure does not indirectly affect song sparrow trill performance through alterations in body condition, but instead may be affecting trill performance more directly, perhaps through neurological or endocrine routes. Considering that much of song sparrow song variability is embedded in trill type and note complexity [48], this relationship between mono-*ortho* PCB blood concentrations and trill performance is a significant finding, and suggests that song sparrows are exposed to PCBs at a high enough concentration to potentially affect song characteristics.

Male song quality and body condition often covary across species [24], [25]. However, they did not covary in the black-capped chickadees and the song sparrows of this study. Only tarsus length significantly varied between regions in the black-capped chickadee sample, with individuals from the ++ Hudson region having significantly longer tarsi than individuals from the − Adirondacks and − Hudson regions. While a longer tarsus might indicate better body condition, our results indicate that black-capped chickadees in the ++ Hudson region do not have ‘better’ songs. Song sparrows differed significantly in two measures of body condition, muscle score and fat score. Taken together, there is some support that individuals in the + Hudson and ++ Hudson regions are in better condition that in the other regions, which could influence trill performance. Yet when analyzed with GLMs and AIC ranking, the body condition index (tarsus/mass) was not the most important predictor for trill performance. Therefore, although components of size and conditions vary between regions, they do not seem to fully explain the song variation in either black-capped chickadees or song sparrows. Putative effects of PCB on song are therefore likely to be direct.

In addition, it is important to consider that PCBs may not be the only pollutant in these study regions affecting birdsong. Mercury is a pollutant in New York State, specifically in the − Adirondacks region (e.g. [26], [49]), and is a neurotoxin known to impair a variety of motor and cognitive abilities [50], including birdsong [51], [52]. Song sparrows had significantly higher blood mercury concentrations in the − Adirondacks compared to all other regions except − Hudson (Figure S4) and exposure to mercury and PCBs seems to minimally overlap in song sparrows between study regions (Figure 2A and Figure S4). The song sparrow blood mercury concentrations were approximately one order of magnitude less than reported from common loons (*Gavia immer*) from the same region [53]. This difference in blood mercury concentrations between song sparrows and common loons is most likely explained by trophic level differences, since mercury biomagnifies and the common loon is an obligate fish eater [53], [54]. The level of mercury measured in the song sparrows was also approximately one order of magnitude lower than concentrations found in studies where birdsong was affected [51], [52]. Therefore, it is unlikely that mercury exposure is confounding trill performance in song sparrows in our study regions.

Results from this study suggest that black-capped chickadees are singing a disrupted species signal in areas of environmental PCB contamination, and that variation in song sparrow trill performance is best predicted by the amount of mono-*ortho* PCBs in an individual. The higher chlorinated PCB congeners that are found predominantly in black-capped chickadees might have detrimental effects on the song system. This could result in the inability of a higher proportion of individuals living in PCB contaminated regions to sing stereotyped glissando ratios. Since song sparrows in ++ Hudson region have the highest PCB levels (Figure 2A) and the level of mono-*ortho* PCB congeners in an individual best predict trill performance (Table 1), we speculate that the large proportion of high trill performance singers (Figure 4B) may be a result of PCBs acting as hormone mimics to increase trill performance. This has been previously seen in European starlings (*Sturnus vulgaris*), where song complexity and the volume of a song production brain nucleus, the HVC, increased with synthetic and natural estrogen exposure [19].

Although the critical consequences of PCB exposure have been broadly explored, the behavioral effects of lower PCB levels in the field are not yet resolved. To our knowledge, this is the first study to investigate the effect of sublethal PCB-exposure on birdsong in the field. Our results show that non-lethal sampling methods are successful in quantifying congener-specific PCB levels in the blood of wild passerines. Furthermore, we show that the behavioral endpoint of birdsong varies significantly in areas of PCB contamination, suggesting that sublethal levels of PCBs may affect social communication. We suggest that future studies should record song and measure PCB congener composition in the same individual (as we were able to do for song sparrows), rather than average across individuals at the same site, for increased clarity of result interpretation.

PCBs have been implicated in behavioral changes in human populations [55]. Therefore, in conjunction with our results, the available data indicate that further research investigating the behavioral effects of sublethal PCBs across multiple taxa is an important step in fully understanding the consequences of this chemical pollutant. We specifically suggest expanding field studies to better understand how suites of different PCB congeners effect behavior, and laboratory research to establish a causal relationship between sublethal dietary PCB levels and behavioral changes, including song.

## Supporting Information

Figure S1 **Black-capped chickadee spectrograms.** The glissando ratio is the frequencyA/frequencyB. The interval ratio is the frequencyB/frequencyC. **A**) Spectrogram of a male black-capped chickadee *fee-bee* song recorded in 2006 from − Adirondacks with a relatively high glissando ratio (1.45) and low interval ratio (0.83). **B**) Spectrogram of a male black-capped chickadee *fee-bee* song recorded in 2007 from + Hudson with a relatively low glissando ratio (1.05) and high interval ratio (1.11).
(TIFF)

Figure S2 **Song sparrow spectrograms.** The trill rate (TR) is the number of syllables/unit time. The bandwidth (BW) is the span of frequencies (high-low) of a syllable. **A**) Spectrogram of a male song sparrow song type recorded in 2007 from − Ithaca with a relatively low performance trill (distance 5.53 kHz from the regression line). **B**) Spectrogram of a male song sparrow song type recorded in 2007 from − Adirondacks with a relatively high performance trill (distance 0.20 kHz from the regression line).
(TIFF)

Figure S3 **Examples of stereotyped and variable glissando and interval ratios of black-capped chickadees from recorded individuals.**
(TIFF)

Figure S4 **Mercury concentrations in song sparrow blood collected in 2008.** Bars are mean±SE, sample size for each region is in the bars, and non-overlapping letters indicate statistical difference between the regions.
(TIFF)

Table S1 Individual PCB congener concentrations. Concentrations are shown as average (ppb) ±SE for black-capped chickadees (**A**) and song sparrows (**B**), by region. The limit of quantification (LOQ) and the limit of detection (LOD) are given for each congener (**C**).
(DOCX)

Table S2 Black-capped chickadee glissando and interval ratios by region. Ratios are shown as mean±SD. Percent deviation is calculated from published ratio values in Christie *et al.* [43].
(DOCX)
