# Supplementary figures and images for: The Effect of Polychlorinated Biphenyls on the Song of Two Passerine Species
Source: PLoS One. 2013 Sep 18;8(9):e73471. doi: 10.1371/journal.pone.0073471 (PMC3776824; doi:10.1371/journal.pone.0073471)

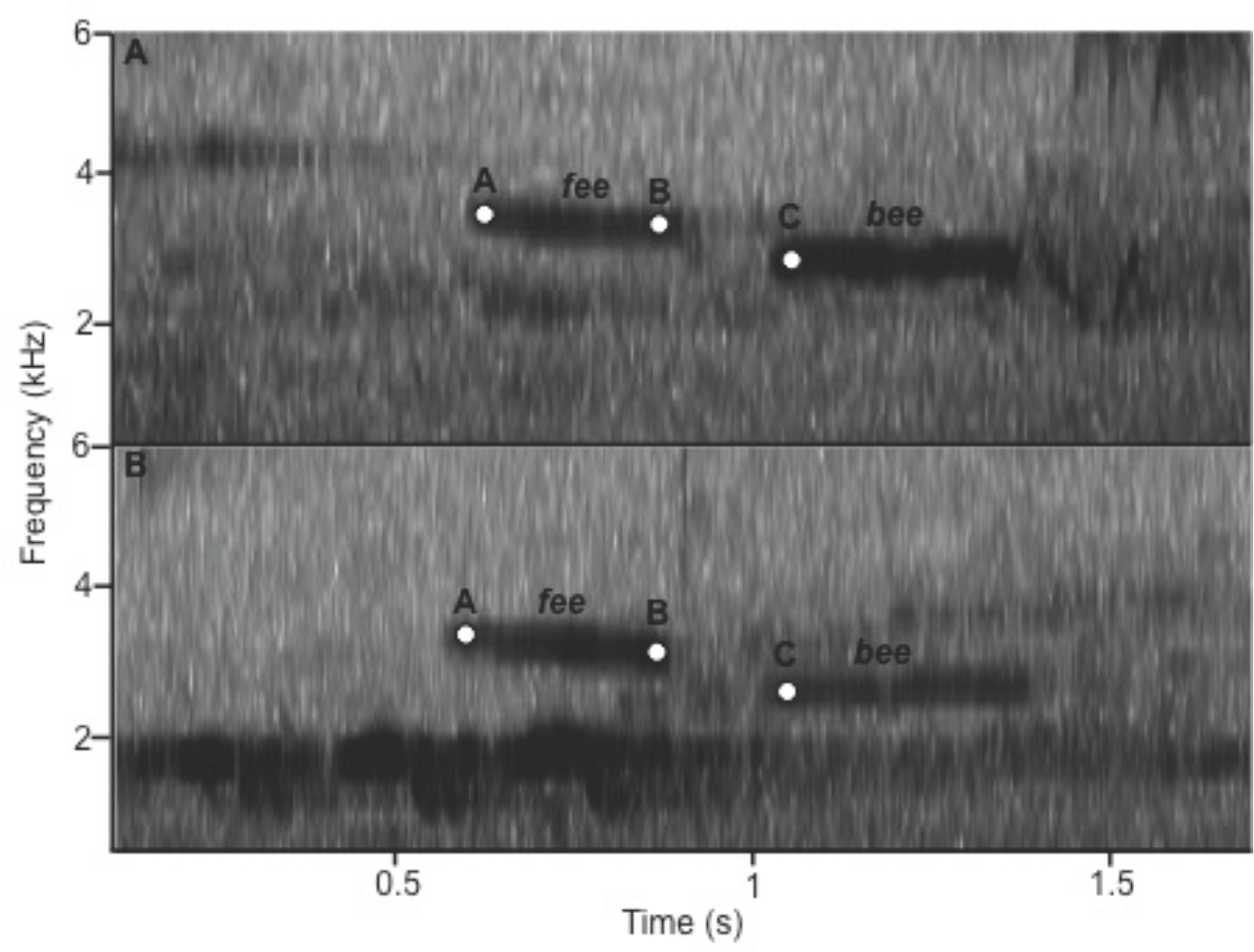

Supplement: Figure S1 — Black-capped chickadee spectrograms. The glissando ratio is the frequencyA/frequencyB. The interval ratio is the frequencyB/frequencyC. A) Spectrogram of a male black-capped chickadee fee-bee song recorded in 2006 from − Adirondacks with a relatively high glissando ratio (1.45) and low interval ratio (0.83). B) Spectrogram of a male black-capped chickadee fee-bee song recorded in 2007 from + Hudson with a relatively low glissando ratio (1.05) and high interval ratio (1.11). (TIFF) [file pone.0073471.s001.tiff]

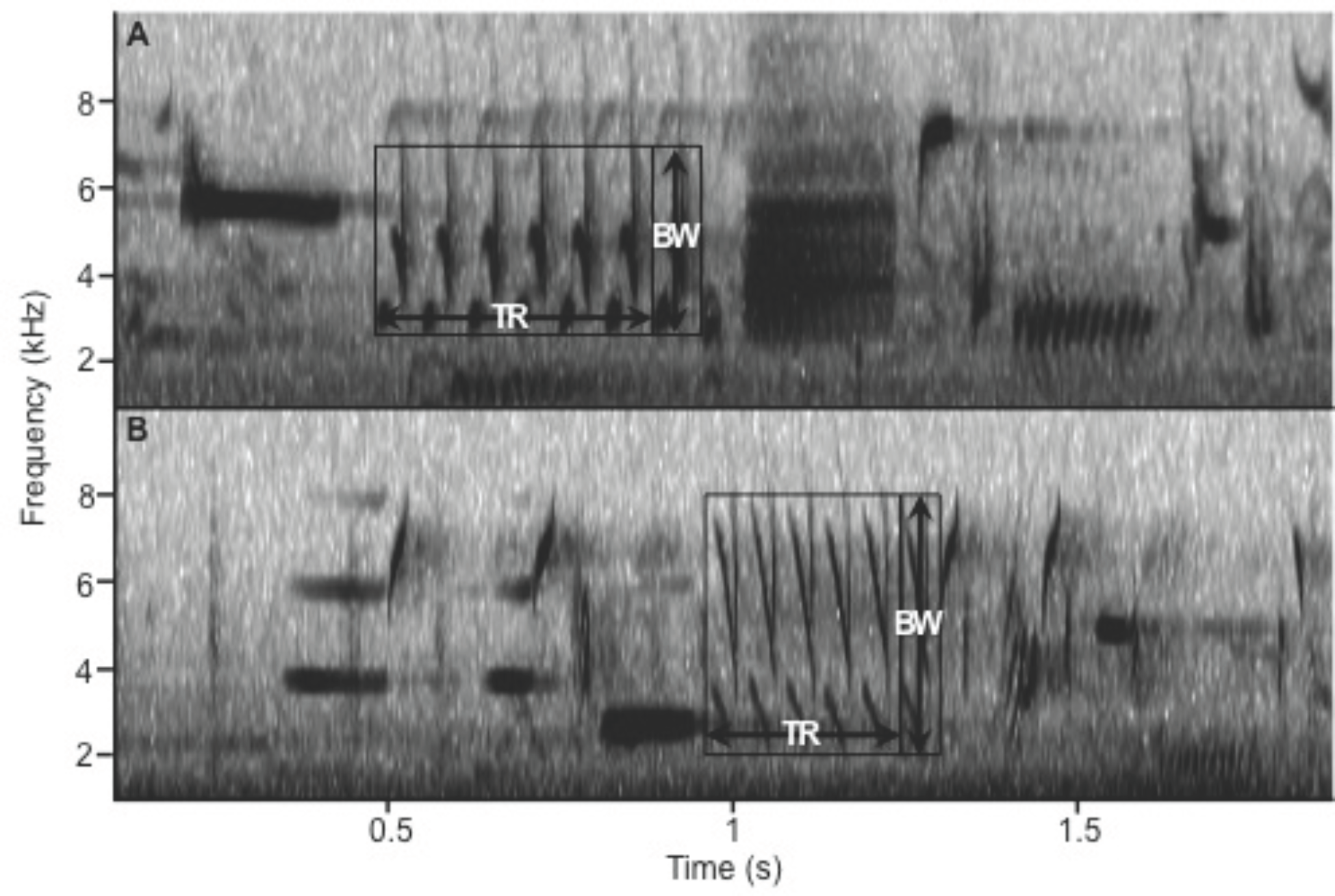

Supplement: Figure S2 — Song sparrow spectrograms. The trill rate (TR) is the number of syllables/unit time. The bandwidth (BW) is the span of frequencies (high-low) of a syllable. A) Spectrogram of a male song sparrow song type recorded in 2007 from − Ithaca with a relatively low performance trill (distance 5.53 kHz from the regression line). B) Spectrogram of a male song sparrow song type recorded in 2007 from − Adirondacks with a relatively high performance trill (distance 0.20 kHz from the regression line). (TIFF) [file pone.0073471.s002.tiff]

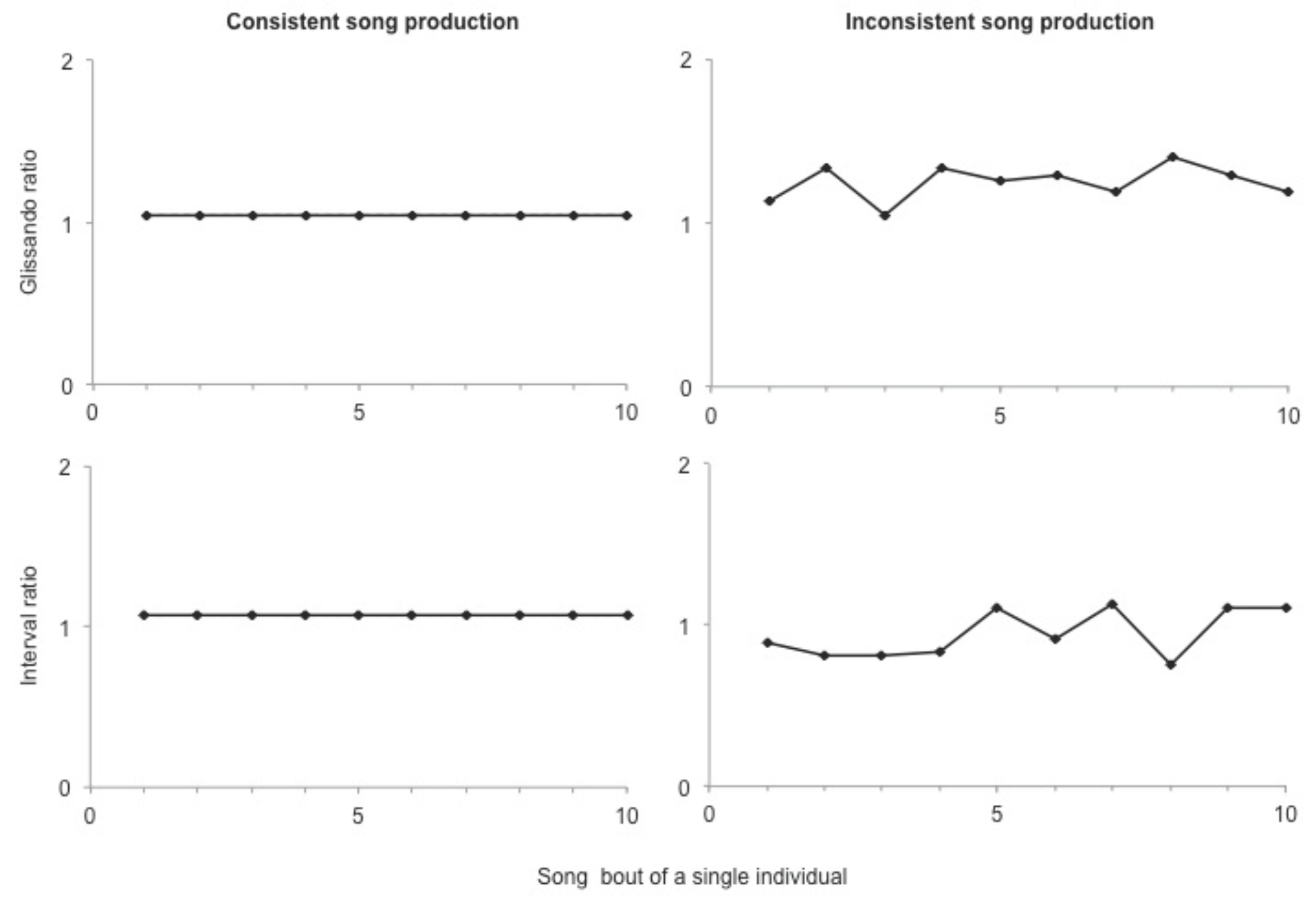

Supplement: Figure S3 — Examples of stereotyped and variable glissando and interval ratios of black-capped chickadees from recorded individuals. (TIFF) [file pone.0073471.s003.tiff]

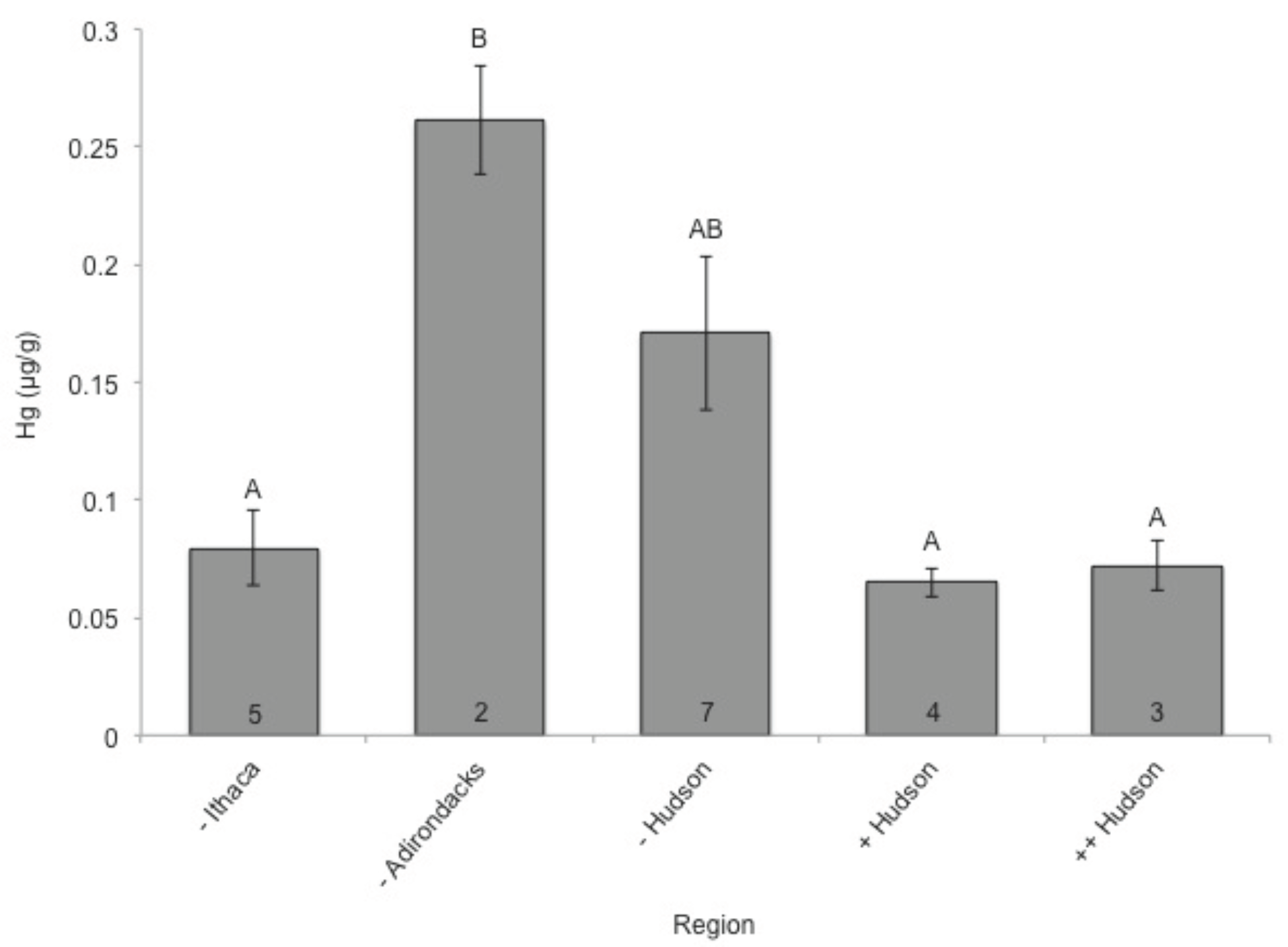

Supplement: Figure S4 — Mercury concentrations in song sparrow blood collected in 2008. Bars are mean±SE, sample size for each region is in the bars, and non-overlapping letters indicate statistical difference between the regions. (TIFF) [file pone.0073471.s004.tiff]
