# Supplementary material for: The Effect of Polychlorinated Biphenyls on the Song of Two Passerine Species
Source: PLoS One. 2013 Sep 18;8(9):e73471. doi: 10.1371/journal.pone.0073471 (PMC3776824; doi:10.1371/journal.pone.0073471)
Supplement: Table S1 — Individual PCB congener concentrations. Concentrations are shown as average (ppb) ±SE for black-capped chickadees (A) and song sparrows (B), by region. The limit of quantification (LOQ) and the limit of detection (LOD) are given for each congener (C). (DOCX) [file pone.0073471.s005.docx]

| **A)** Black-capped chickadees | | | | | | |
| --- | --- | --- | --- | --- | --- | --- |
|  | Region | - Ithaca | - Adirondacks | - Hudson | + Hudson | ++ Hudson |
| PCB congener | 8 | 0±0 | 0±0 | 0±0 | 0±0 | 0±0 |
|  | 18 | 0±0 | 0±0 | 0±0 | 0±0 | 0±0 |
|  | 28/31 | 0±0 | 0±0 | 0.059±0.043 | 0±0 | 3.241±0.86 |
|  | 44 | 0±0 | 0±0 | 0±0 | 0±0 | 0.122±0.094 |
|  | 45 | 0±0 | 0±0 | 0±0 | 0±0 | 0±0 |
|  | 47 | 0±0 | 0±0 | 0.132±0.085 | 0.973±0.487 | 18.104±2.592 |
|  | 49 | 0±0 | 0±0 | 0±0 | 0.183±0.183 | 8.208±1.783 |
|  | 52 | 0±0 | 0±0 | 0±0 | 0.577±0.31 | 16.975±2.986 |
|  | 56/60 | 0.21±0.21 | 0.197±0.197 | 1.226±0.549 | 1.96±1.96 | 3.938±0.996 |
|  | 66 | 0.196±0.196 | 0±0 | 1.553±0.428 | 0.897±0.491 | 18.311±3.285 |
|  | 70 | 0.396±0.246 | 0.25±0.25 | 1.392±0.574 | 0.197±0.197 | 0.759±0.247 |
|  | 74 | 0.246±0.215 | 0±0 | 1.779±0.577 | 2.897±1.128 | 16.191±3.265 |
|  | 77 | 0±0 | 0±0 | 0.257±0.257 | 0±0 | 0±0 |
|  | 81 | 0±0 | 0±0 | 0±0 | 0±0 | 0±0 |
|  | 87 | 0±0 | 0±0 | 0±0 | 0±0 | 1.105±0.483 |
|  | 95 | 2.342±1.609 | 2.103±1.112 | 7.784±4.704 | 1.827±1.827 | 0±0 |
|  | 105 | 0±0 | 0±0 | 1.065±0.794 | 0±0 | 12.133±2.172 |
|  | 110 | 0±0 | 0±0 | 0±0 | 0±0 | 0.377±0.377 |
|  | 114 | 0±0 | 0.885±0.885 | 0±0 | 0±0 | 1.39±0.584 |
|  | 118 | 0±0 | 0±0 | 2.386±1.173 | 2.317±2.317 | 30.018±7.899 |
|  | 123 | 0.196±0.196 | 0±0 | 0±0 | 0±0 | 1.629±0.494 |
|  | 128 | 0±0 | 0±0 | 0.059±0.059 | 0.553±0.279 | 9.628±2.962 |
|  | 132/153 | 2.436±0.819 | 1.09±0.37 | 9.457±2.861 | 35.843±6.634 | 82.425±15.824 |
|  | 146 | 0±0 | 0±0 | 1.159±0.413 | 6.417±1.27 | 14.195±2.755 |
|  | 149 | 0±0 | 0±0 | 0.218±0.12 | 0±0 | 5.594±1.241 |
|  | 151 | 0±0 | 0±0 | 0.038±0.038 | 0±0 | 0.368±0.216 |
|  | 156 | 0±0 | 0±0 | 0.148±0.148 | 1.083±0.545 | 4.746±1.313 |
|  | 157 | 0±0 | 0±0 | 0±0 | 0±0 | 1.108±0.431 |
|  | 167 | 0±0 | 0.617±0.617 | 0±0 | 0±0 | 1.723±0.907 |
|  | 169 | 0±0 | 0±0 | 0±0 | 0±0 | 0.254±0.254 |
|  | 170 | 3.26±1.777 | 0.345±0.345 | 2.831±0.843 | 5.497±0.924 | 12.391±3.105 |
|  | 174 | 0.288±0.288 | 0±0 | 0.207±0.131 | 1.11±0.681 | 1.555±1.221 |
|  | 177 | 1.16±1.16 | 0±0 | 0.077±0.06 | 0±0 | 1.26±0.952 |
|  | 180 | 2.06±1.466 | 0.525±0.525 | 5.891±2.641 | 22.767±4.168 | 41.49±15.011 |
|  | 183 | 1.88±1.491 | 0±0 | 1.988±1.039 | 4.1±3.489 | 16.779±9.478 |
|  | 187 | 0.334±0.334 | 0.665±0.665 | 0.96±0.75 | 0±0 | 19.227±19.227 |
|  | 194 | 0±0 | 0±0 | 0.686±0.579 | 0.833±0.833 | 5.195±1.344 |
|  | 195 | 0±0 | 0±0 | 0±0 | 0±0 | 0.558±0.373 |
|  | 206 | 0.974±0.601 | 0.34±0.34 | 0.719±0.248 | 1.827±0.982 | 3.124±0.774 |
|  | 209 | 0±0 | 0±0 | 0±0 | 0±0 | 0±0 |
|  | Total | 15.98±5.432 | 5.843±2.74 | 41.578±9.455 | 91.85±2.816 | 354.122±74.389 |
|  | N | 5 | 3 | 18 | 3 | 10 |

| **B)** Song sparrows | | | | | | |
| --- | --- | --- | --- | --- | --- | --- |
|  | Region | - Ithaca | - Adirondacks | - Hudson | + Hudson | ++ Hudson |
| PCB congener | 8 | 0±0 | 0±0 | 0±0 | 0±0 | 0±0 |
|  | 18 | 0±0 | 0±0 | 0±0 | 0±0 | 0±0 |
|  | 28/31 | 0±0 | 0±0 | 0.723±0.207 | 4.427±0.842 | 89.838±18.257 |
|  | 44 | 0±0 | 0±0 | 0±0 | 0±0 | 0.071±0.047 |
|  | 45 | 0±0 | 0±0 | 0±0 | 0±0 | 0±0 |
|  | 47 | 0.254±0.13 | 0.171±0.072 | 0.741±0.134 | 5.997±0.929 | 121.378±21.586 |
|  | 49 | 0±0 | 0±0 | 0.031±0.031 | 0.568±0.144 | 26.247±4.586 |
|  | 52 | 0±0 | 0±0 | 0±0 | 0.288±0.122 | 22.021±4.317 |
|  | 56/60 | 0±0 | 0±0 | 0.263±0.087 | 0.536±0.153 | 36.893±8.839 |
|  | 66 | 0.736±0.315 | 0.57±0.214 | 3.293±0.663 | 5.592±0.752 | 183.613±37.349 |
|  | 70 | 0±0 | 0±0 | 0±0 | 0.284±0.153 | 13.684±2.385 |
|  | 74 | 0.569±0.234 | 0.448±0.113 | 1.92±0.359 | 3.988±0.589 | 112.947±23.616 |
|  | 77 | 0±0 | 0±0 | 0±0 | 0.095±0.055 | 0.281±0.084 |
|  | 81 | 0±0 | 0±0 | 0±0 | 0±0 | 0.113±0.082 |
|  | 87 | 0±0 | 0±0 | 0±0 | 0±0 | 0.812±0.595 |
|  | 95 | 0±0 | 0±0 | 0.138±0.138 | 0±0 | 0±0 |
|  | 105 | 0±0 | 0.445±0.319 | 1.653±0.359 | 3.589±0.488 | 66.555±13.222 |
|  | 110 | 0±0 | 0±0 | 0±0 | 0.739±0.244 | 10.731±2.965 |
|  | 114 | 0±0 | 0.165±0.165 | 0.616±0.27 | 0.771±0.273 | 7.936±1.411 |
|  | 118 | 0.25±0.25 | 1.267±0.654 | 3.242±0.571 | 6.801±1.217 | 98.471±17.874 |
|  | 123 | 0.088±0.088 | 0.055±0.055 | 0.137±0.053 | 0.508±0.161 | 9.203±1.752 |
|  | 128 | 0.044±0.044 | 0.056±0.056 | 0.642±0.175 | 2.279±0.566 | 13.953±3.034 |
|  | 132/153 | 1.54±0.356 | 2.462±0.233 | 8.498±1.327 | 11.486±3.671 | 139.162±20.633 |
|  | 146 | 0±0 | 0±0 | 1.178±0.266 | 1.835±0.583 | 23.431±3.302 |
|  | 149 | 0±0 | 0±0 | 0.036±0.036 | 0.702±0.21 | 8.597±1.472 |
|  | 151 | 0±0 | 0±0 | 0±0 | 0±0 | 0.255±0.093 |
|  | 156 | 0.106±0.073 | 0±0 | 0.297±0.089 | 0.563±0.141 | 4.773±0.663 |
|  | 157 | 0±0 | 0±0 | 0±0 | 0.083±0.058 | 2.003±0.421 |
|  | 167 | 0±0 | 0.104±0.104 | 0.422±0.231 | 1.833±0.674 | 2.456±0.632 |
|  | 169 | 0±0 | 0±0 | 0±0 | 0.046±0.046 | 0.035±0.035 |
|  | 170 | 0.576±0.393 | 0.49±0.244 | 2.473±0.657 | 8.979±2.207 | 25.404±5.562 |
|  | 174 | 0±0 | 0±0 | 0.027±0.027 | 0.219±0.102 | 0±0 |
|  | 177 | 0.023±0.023 | 0±0 | 1.398±0.412 | 0.91±0.342 | 16.8±5.708 |
|  | 180 | 2.552±1.019 | 1.491±0.483 | 9.424±1.99 | 18.731±4.489 | 94.144±23.971 |
|  | 183 | 0.691±0.366 | 0.734±0.366 | 4.284±1.418 | 3.455±1.173 | 42.868±15.159 |
|  | 187 | 0±0 | 0.107±0.107 | 0±0 | 2.745±0.698 | 0±0 |
|  | 194 | 0.887±0.435 | 0.797±0.293 | 1.616±0.425 | 2.1±0.381 | 5.119±0.569 |
|  | 195 | 0.729±0.426 | 0.635±0.407 | 0.568±0.195 | 1.303±0.415 | 1.749±0.322 |
|  | 206 | 0.785±0.528 | 0.646±0.247 | 0.772±0.191 | 3.442±0.67 | 8.007±1.603 |
|  | 209 | 0±0 | 0.742±0.742 | 0±0 | 0.156±0.156 | 0.168±0.168 |
|  | Total | 9.831±2.788 | 10.835±1.444 | 42.294±6.359 | 95.011±13.712 | 1150.065±185.934 |
|  | N | 14 | 11 | 29 | 32 | 39 |

| **C)** | | LOQ | LOD |
| --- | --- | --- | --- |
| PCB congener | 8 | 0.5 | 0.1 |
|  | 18 | 0.5 | 0.1 |
|  | 28/31 | 0.25 | 0.1 |
|  | 44 | 0.1 | 0.1 |
|  | 45 | 0.1 | 0.1 |
|  | 47 | 0.25 | 0.1 |
|  | 49 | 0.5 | 0.1 |
|  | 52 | 0.5 | 0.1 |
|  | 56/60 | 0.5 | 0.25 |
|  | 66 | 0.5 | 0.1 |
|  | 70 | 0.5 | 0.1 |
|  | 74 | 0.1 | 0.1 |
|  | 77 | 0.5 | 0.25 |
|  | 81 | 1 | 0.25 |
|  | 87 | 1 | 0.25 |
|  | 95 | 1 | 0.25 |
|  | 105 | 1 | 0.1 |
|  | 110 | 1 | 0.25 |
|  | 114 | 0.25 | 0.1 |
|  | 118 | 0.5 | 0.25 |
|  | 123 | 0.1 | 0.1 |
|  | 128 | 0.5 | 0.25 |
|  | 132/153 | 0.25 | 0.1 |
|  | 146 | 1 | 0.1 |
|  | 149 | 1 | 0.1 |
|  | 151 | 0.5 | 0.25 |
|  | 156 | 0.5 | 0.1 |
|  | 157 | 1 | 0.25 |
|  | 167 | 1 | 0.25 |
|  | 169 | 1 | 0.25 |
|  | 170 | 2.5 | 0.1 |
|  | 174 | 0.5 | 0.25 |
|  | 177 | 0.5 | 0.25 |
|  | 180 | 2.5 | 0.25 |
|  | 183 | 0.5 | 0.25 |
|  | 187 | 0.5 | 0.25 |
|  | 194 | 1 | 0.1 |
|  | 195 | 1 | 0.1 |
|  | 206 | 1 | 0.1 |
|  | 209 | 1 | 1 |
