# Supplementary material for: The Effect of Polychlorinated Biphenyls on the Song of Two Passerine Species
Source: PLoS One. 2013 Sep 18;8(9):e73471. doi: 10.1371/journal.pone.0073471 (PMC3776824; doi:10.1371/journal.pone.0073471)
Supplement: Table S2 — Black-capped chickadee glissando and interval ratios by region. Ratios are shown as mean±SD. Percent deviation is calculated from published ratio values in Christie et al. [43]. (DOCX) [file pone.0073471.s006.docx]

|  | Glissando ratio | | | Interval ratio | | |
| --- | --- | --- | --- | --- | --- | --- |
| **Region** | **Sample Size** | **Average Glissando Ratio±SD** | **% from published value** | **Sample Size** | **Average Interval Ratio±SD** | **% from published value** |
| - Ithaca | 38 | 1.067±0.017 | 1.02 | 38 | 1.137±0.017 | 0.25 |
| - Adirondacks | 70 | 1.073±0.016 | 1.63 | 78 | 1.139±0.020 | 0.42 |
| - Hudson | 23 | 1.074±0.023 | 1.70 | 23 | 1.157±0.018 | 2.07 |
| + Hudson | 39 | 1.123±0.058 | 6.36 | 39 | 1.131±0.015 | -0.24 |
| ++ Hudson | 40 | 1.091±0.033 | 3.27 | 40 | 1.142±0.019 | 0.70 |
